# Redefining Medical Publishing in the Artificial Intelligence Era

**DOI:** 10.1002/hcs2.70026

**Published:** 2025-07-30

**Authors:** You Wu, Haibo Wang

**Affiliations:** ^1^ School of Basic Medical Sciences, Tsinghua Medicine, Institute for Hospital Management Tsinghua University Beijing China; ^2^ Research Centre of Big Data and Artificial Research for Medicine, The First Affiliated Hospital Sun Yat‐sen University Guangzhou China

**Keywords:** AI, data sharing, medical data, medical publishing

On April 26, 2025, the Second Tsinghua Medicine Journal Innovation Conference convened in Beijing. Centered on the theme “AI‐driven Academic: Shaping the Next Frontier” the Conference brought together journal editors, medical researchers, and science policy experts to examine how data and artificial intelligence (AI) are reshaping scholarly publishing. Two keynote speeches set the stage: the first analyzed the opportunities for hospital‐based research arising from new journal policies, data infrastructure, and enabling technologies; the second introduced the latest advances in general AI and their implications for academic publishing security and integrity.

The conference unfolded through four roundtable discussions, each addressing critical intersections of AI and medical publishing. The first session explored strategic approaches to hospital research planning and AI's catalytic role in medical innovation. The second examined how academic journals can leverage AI to enhance editorial workflows and amplify the global influence of Chinese research. The third delved into institutional strategies for building interdisciplinary research clusters, with journals serving as key dissemination platforms. The concluding discussion identified systemic bottlenecks in translational research while championing cross‐sector collaboration to bridge the gap between laboratory discoveries and clinical applications. Together, these dialogues mapped the complex ecosystem of challenges and solutions reshaping medical knowledge dissemination.

AI's transformative impact manifests across healthcare's clinical and academic dimensions. Beijing Children's Hospital, Capital Medical University demonstrated their “Futang Baichuan” pediatric AI system—A state‐of‐the‐art model trained on 38 million research publications, 40,000+ clinical guidelines, and 80 years of institutional case data [1]. Beyond achieving 95% diagnostic concordance with senior specialists, its dedicated research module autonomously generates hypotheses by mining multimodal clinical data, creating a closed‐loop system between bedside practice and bench research.

The publishing workflow is also being rapidly transformed by AI. Elsevier has launched its end‐to‐end Research Information Management System (RIMS) product, integrating literature management and data storage, boosting efficiency by 40% [2]. As for the scientific editing process, Dr. Yong Hu introduced a pilot study where large language models could complete most of pre‐screening and basic editing tasks, albeit with a very low rate of hallucinated errors. AgentReview, a novel LLM‐based simulation framework to analyze peer review dynamics, revealed a 37.1% decision variance due to reviewer biases while addressing privacy concerns and latent factors in the process [3].

As AI development accelerates exponentially, more people began to recognize the value of its true enabler—data. Medical data are no longer a mere byproduct of research but a critical asset. The explosive growth of data papers supports this shift. Data papers—also known as data descriptors or data set articles—are scholarly publications that focus on the detailed description, validation, and potential reuse of datasets rather than traditional hypothesis‐driven results. These papers typically include comprehensive metadata, data collection and processing methods, and usage notes to facilitate reproducibility. Examples include the *Data in Brief* journal [4] by Elsevier and Scientific Data [5] by *Nature*. According to Web of Science, over 4800 data papers in the medical field had been published by early 2025, collectively cited 241,000 times—an average of 5.02 citations per article, compared to 2.8 for traditional papers. Leading publishers like Elsevier and SpringerNature encourage authors to deposit their supporting data in publicly available repositories, or reporting this within the manuscript or additional supporting files. Meanwhile, the International Association of Scientific, Technical and Medical Publishers has published “12 Best Practices for Data Sharing,” proposing new standards for assessing academic impact [6]. Notably, data reuse is emerging as a key metric, signaling a shift from static publications to dynamic, infrastructure‐based science.

The importance of controlling access to biomedical data became clear earlier this month, when the National Institutes of Health suddenly restricted China's access to some of its major biomedical databases. This move highlighted the value of China's decision—made 10 years earlier—to launch the “Scientific Data Bank” initiative through the Chinese Academy of Sciences. Between 1989 and 2017, use of the NCBI databases grew rapidly, with Chinese researchers accounting for about 15% of all visits. The new restrictions hit Chinese biomedical research hard, especially projects that depend on international open data. However, China does have its health data sharing initiatives and measures in advance to cope with such unforeseeable challenges [7, 8]. It also underscored why the Ministry of Science and Technology of the People's Republic of China, back in 2018, began treating scientific data as a national strategic resource through its “Measures for the Management of Scientific Data” So far, China has established 20 national scientific data centers, including the Population Health Data Archive. For projects sponsored by the National Health Commission of the People's Republic of China, the data deposition rate has reached 100%.

During the round table sessions, there was strong support among the participants for establishing a national alliance of disease‐specific medical databases to address the problem of fragmented data resources. Second, participants advocated for incorporating data papers into professional evaluation systems, including the possibility of adding credit for such publications during academic promotions. Third, while proposing AI‐powered prescreening workflows, there was a consensus that the tool should be able to run locally (instead of on cloud), and that the final editorial decisions should always rely on human experts. Finally, the construction of a sovereign and interoperable academic infrastructure was identified as a long‐term strategic goal to help China develop globally competitive scientific publishing platforms.

Driven by growing geopolitical uncertainties and rapid advances in AI and data infrastructure, the medical publishing sector stands at a crossroads. At the conference, participants shared how institutions are responding—with better data strategies, AI‐supported editorial systems, and improved national platforms. These shared priorities may signal a new phase in the development of medical publishing, with lasting effects on global research.

## Author Contributions

**You Wu:** conceptualization (equal), writing – review and editing (equal). **Haibo Wang:** conceptualization (equal), resources (equal).

## Ethics Statement

The authors have nothing to report.

## Consent

The authors have nothing to report.

## Conflicts of Interest

Professor Haibo Wang is a member of the *Health Care Science* Editorial Board. To minimize bias, he was excluded from all editorial decision‐making related to the acceptance of this article for publication. The remaining author declares no conflicts of interest.

## Data Availability

The authors have nothing to report.
